# ATOM Calibration Framework: Interaction and Visualization Functionalities

**DOI:** 10.3390/s23020936

**Published:** 2023-01-13

**Authors:** Manuel Gomes, Miguel Oliveira, Vítor Santos

**Affiliations:** 1 Intelligent System Associate Laboratory (LASI), Institute of Electronics and Informatics Engineering of Aveiro (IEETA), University of Aveiro, 3810-193 Aveiro, Portugal; 2 Department of Mechanical Engineering, University of Aveiro, 3810-193 Aveiro, Portugal

**Keywords:** extrinsic calibration, intrinsic calibration, user experience, multi-modal, multi-sensor, ROS

## Abstract

Robotic systems are evolving to include a large number of sensors and diverse sensor modalities. In order to operate a system with multiple sensors, the geometric transformations between those sensors must be accurately estimated. The process by which these transformations are estimated is known as sensor calibration. Behind every sensor calibration approach is a formulation and a framework. The formulation is the method by which the transformations are estimated. The framework is the set of operations required to carry out the calibration procedure. This paper proposes a novel calibration framework that gives more flexibility, control and information to the user, enhancing the user interface and the user experience of calibrating a robotic system. The framework consists of several visualization and interaction functionalities useful for a calibration procedure, such as the estimation of the initial pose of the sensors, the data collection and labeling, the data review and correction and the visualization of the estimation of the extrinsic and intrinsic parameters. This framework is supported by the Atomic Transformations Optimization Method formulation, referred to as ATOM. Results show that this framework is applicable to various robotic systems with different configurations, number of sensors and sensor modalities. In addition to this, a survey comparing the frameworks of different calibration approaches shows that ATOM provides a very good user experience.

## 1. Introduction

Recent years have shown that the development of robotic and intelligent systems is paired with an increase in the number of sensors and sensor modalities installed in the systems. These systems with enhanced intelligence operate by processing data from several sensors and modalities. The usage of multiple sensor sources creates the need to transform data between the coordinate systems of the sensors. To accomplish this, the geometric transformations between the sensors need to be accurately estimated. The process by which those transformations are estimated is called sensor calibration.

Sensor calibration can be divided into three different variants [1], depending on the configuration of the system. The classic one is called sensor to sensor. In this variant, the objective is to retrieve the transformation of one sensor with regard to another. Some examples are RGB camera to RGB camera calibration [2,3] and Light Detection And Ranging (LiDAR) to LiDAR calibration [4,5]. The second variant, sensor in motion, refers to the case of a moving sensor, and the objective is to estimate the transformations between the different poses of the sensor over time. Some examples are Simultaneous Localization and Mapping (SLAM) [6] and Structure From Motion (SFM) [7]. The third variant, sensor to frame, aims to estimate the transformation between a sensor and a coordinate frame of interest. Some examples are the hand–eye calibration [8] and the RGB camera to robot frame calibration [9].

In addition to these variants, the sensor calibration problem can be further extended by adding multiple sensors. We refer to this as multi-sensor calibration. Examples are a system with three different LiDAR sensors [10] and a network system of cameras [11]. Moreover, it is also possible to combine sensors of multiple modalities. We refer to this as multi-modal calibration. Examples are the RGB camera to depth camera calibration [12] and the RGB camera to LiDAR calibration [13].

A calibration methodology is crucial to conduct accurate calibrations. However, a methodology is insufficient to carry out a successful calibration, as it needs to receive labeled sensor data, evaluate the calibration and so on. To tackle all these problems in an integrated fashion, a complete calibration framework must be developed. The framework must address these issues and provide visualization and interactive functionalities to allow the user to inspect and configure the calibration procedure. Although at first glance one may think that a calibration procedure should be fully automatic, in practice, there is always the need to conduct several manual operations, such as the inspection and possibly the correction of labeled data. As such, one crucial part of the framework is the User Interface (UI) and User Experience (UX).

Most of the existing calibration frameworks are focused on the calibration methodology and have minimal UIs. Some approaches do not provide ways for collecting or labeling the data. These solutions are sufficient for simple robotic systems, such as a stereo camera. However, in the case of complex multi-sensor, multi-modal systems, the collection of data can be cumbersome. The labeling of data is often carried out automatically. This is possible especially for RGB sensors. However, it is important to be able to review the labeling of data, as it is crucial to the accuracy of the produced estimations. While some approaches provide a way to inspect the labeling, there is no possibility to manually correct the automatically produced labels. Another missing aspect in most calibration frameworks is the visualization component. In fact, several approaches conduct the estimation of geometric transformations between sensors without conveying visual feedback to the user.

Since the focus of this paper is on the calibration of complex robotic systems, we view the integration of our calibration framework with Robot Operating System (ROS) as a very important aspect. In fact, the developed interfaces and visualization means are all supported by ROS tools. Our previous work focused on the development of the Atomic Transformations Optimization Method’s (ATOM) calibration methodology. In particular, we worked on expanding the methodology for tackling hand–eye calibration problems [14], agricultural robots [15], autonomous vehicles [16] and collaborative cells [17]. In addition, we have shown several examples of successful calibrations [1].

This paper focuses on the user interface and user experience of the ATOM calibration framework. As such, the contributions are the following:

- ATOM, an extended calibration framework integrated into ROS, containing several visualizations and interaction functionalities useful for a calibration procedure, such as:
  - The estimation of the initial pose of the sensors;
  - The data collection and labeling;
  - The data review and correction;
  - The estimation of the extrinsic and intrinsic parameters.
- A survey on the topic of calibration frameworks which shows how visualization and interactive functionalities are helpful for roboticists.

The contributions listed above are novel because most calibration approaches have lackluster or nonexistent support for these functionalities, relying on non-intuitive or external tools with minimal or absent graphical UIs for these functionalities.

This paper is structured into five different sections. The Introduction section (Section 1) discusses the purpose of this work, as well as previous work on the ATOM calibration framework. The Related Work section (Section 2) describes other calibration frameworks, depicting their advantages and disadvantage. The Proposed Framework section (Section 3) defines in detail the ATOM framework and its stages, focusing on UI and UX elements. The Results section (Section 4) presents an extensive qualitative demonstration of ATOM and the results of a survey on the topic of calibration frameworks. The Conclusions section (Section 5) summarizes the framework, along with its advantages.

## 2. Related Work

As stated in Section 1, the calibration of a system requires not only a method for the optimization of the poses of the sensors but, in addition to this, several other procedures. These include the collection of data, the labeling of data and others. From the review of the state of the art it is clear that there are three main components of a calibration procedure: data collection, data labeling and the sensor pose estimation. Data collection is the process by which sensor data is acquired. There are several concerns to be tackled in this stage, namely the synchronization of data from different sensors and the selection of adequate moments to acquire the data. The sensor labeling component aims to label the data collected in the first stage. Data labeling is typically modality-specific and must support all sensor modalities included in the system. The procedures can be fully automatic, semi-automatic or manual. To ensure that the correspondences between data are as accurate as possible, it is very common to use a calibration pattern. This provides accuracy but also facilitates the automatic detection procedures. Finally, the third component is where the actual calibration is carried out. Sensor poses are estimated using the associations produced by the data labeling. This can be a closed form or an iterative procedure.

In some approaches, these components may not exist or may be fully automatic. Nonetheless, these components are always required in a calibration procedure, even if they are hidden from the user.

This section presents six of the most relevant calibration frameworks that preceded ATOM. These calibration frameworks are: *Open Source Computer Vision Library (OpenCV) Camera Calibration* [18], *camera calibration*, *robot calibration*, *moveit calibration* [19,20], *Kalibr* [21,22,23,24,25] and *OpenCalib* [26]. Their description will be focused on how every calibration component, UI and UX are handled. The benefits and shortcomings of each approach are also detailed.

The *Open Source Computer Vision Library (OpenCV) Camera Calibration* (https://docs.opencv.org/4.x/dc/dbb/tutorial_py_calibration.html, accessed on 25 November 2022) is a popular calibration framework from the OpenCV library [18]. This approach is designed to operate with two RGB cameras viewing a chessboard pattern. This approach does not include a data acquisition component, since prerecorded images must be provided. The data labeling is done automatically, and the estimation of parameters is carried out by a minimization procedure. This approach estimates the sensor poses, i.e., the extrinsic parameters, in addition to the intrinsic and distortion parameters of each RGB camera. The UI and UX are minimal, having only the possibility of displaying the labeled data overlayed on the images to verify if that procedure was carried out accurately.

**Figure 1:**
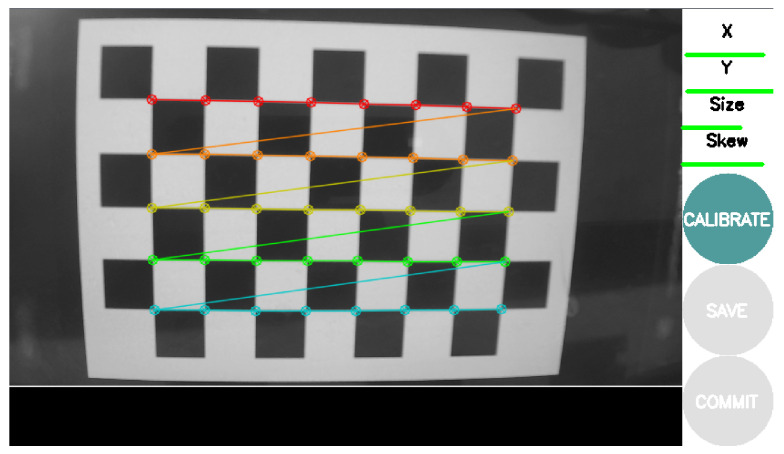
The *camera_calibration* labeling visual feedback is presented on the left, and bars of variation of parameters and user option are represented in buttons shown on the right.

The previous approach [18] was ported to ROS in a tool called *camera_calibration* (https://wiki.ros.org/camera_calibration, accessed on 25 November 2022). As such, it also supports two RGB cameras and a chessboard as the calibration pattern. The addition is a graphical user interface in which the user observes the input image and the result of the labeling, which is carried out in real-time. Data is acquired automatically, using an intelligent algorithm that avoids collecting data similar to previously recorded data. This avoids the creation of very large datasets and also ensures variability in the collected images. The UI presents four bars on the side of the image visualization, which represent the amount of variability already collected for each specific parameter. The user may only proceed with the calibrate option when the system has acquired a set of images containing sufficient variability. The estimation of the parameters is done without any visual feedback (the system freezes when calibrating) and outputs the calibration results to the terminal. The user is then presented with the option to save the parameters inside a *tar.gz* file or to save the parameters to a *yaml*, which is automatically read by the ROS ecosystem. The UI is presented in Figure 1.

One of the most widely used calibration approaches is the *robot_calibration* (https://github.com/mikeferguson/robot_calibration, accessed on 25 November 2022) due to its ROS integration. This approach only supports RGB cameras and also requires a chessboard calibration pattern. Data acquisition can be carried out using two alternatives: manual or automatic. The manual approach relies on the user to decide when the sensor data should be collected. The automatic variant is designed to operate with cameras onboard a robotic manipulator, and the collection of data is triggered automatically when the manipulator reaches each of the predefined waypoints. The estimation of the parameters is done without any visual feedback, and the system outputs the results of the calibration to a Unified Robot Description Format (URDF) and a *yaml* file, which are automatically parsed by the ROS tools.

**Figure 2:**
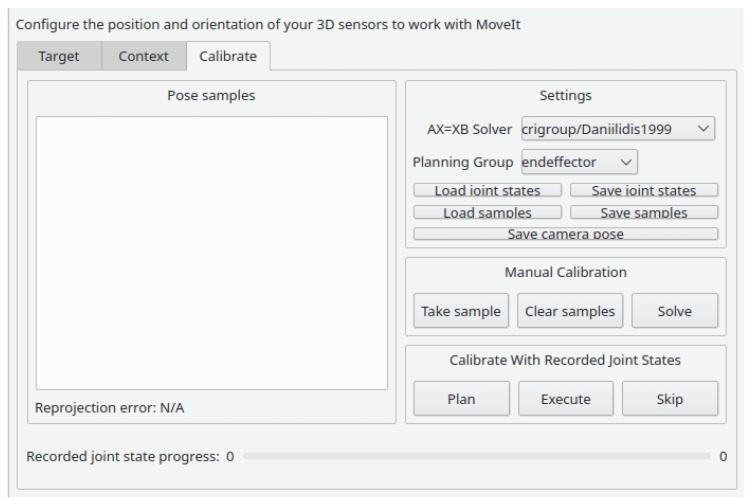
*moveit_calibration* UI inside RViz, where the user can acquire data using the “Take Sample” button and the estimation of parameters carries out automatically [27].

There is also an ROS calibration tool specific for addressing the hand–eye calibration problem called *moveit_calibration* (https://github.com/ros-planning/moveit_calibration, accessed on 25 November 2022) [19,20]. The hand–eye calibration produces an estimate of the transformation between the end effector of a robotic manipulator and a camera mounted on that end effector. It is useful for several applications, such as pick and place or 3D reconstruction. This approach supports only RGB cameras, and the calibration pattern is an ArUco board. The data acquisition and labeling are done simultaneously. This ensures that the user has immediate visual feedback on the produced labels, allowing a more deliberate choice of when to acquire data. As in *robot_calibration*, the data acquisition can be done manually or by pre-planning a path and collecting images on each waypoint. The estimation of the parameters is done without any visual feedback. It outputs an ROS launch file, which publishes the estimated extrinsic parameters as ROS tf static transformations. The UI is based on ROS 3D Robot Visualizer (RViz). The user can define the calibration parameters, acquire data and visualize data labels, as seen in Figure 2. The calibration is done automatically after collecting five data samples and is recomputed each time a new sample is added.

*Kalibr* (https://github.com/ethz-asl/kalibr, accessed on 25 November 2022) is a calibration toolbox integrated into ROS [21,22,23,24,25]. This approach operates with RGB cameras and Inertial Measurement Unit (IMU) sensors and supports the chessboard, AprilTag board (https://april.eecs.umich.edu/software/apriltag, accessed on 25 November 2022) and circlegrid (https://www.mathworks.com/help//vision/ug/calibration-patterns.html, accessed on 25 November 2022) as calibration patterns. In this approach, it is mandatory to prerecord the data in an ROS *bagfile*. This data log is then processed by *Kalibr*, which acquires data automatically. This automatic data labeling usually gathers a large dataset containing hundreds of images. The data labeling is done automatically and is not displayed to the user. The estimation of the parameters is done without visual feedback. The system calibrates both the extrinsic and the intrinsic camera parameters, and outputs the results to a *yaml* file [26]. The UI is practically nonexistent, with all commands given in the terminal. The calibration outputs a *pdf* report containing several statistics and all as charts analyzing the calibration errors [28].

**Figure 3:**
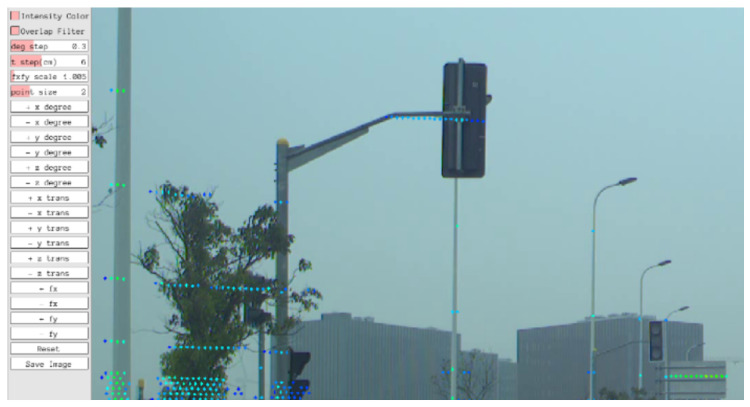
*OpenCalib* UI when calibrating an RGB w.r.t. a LiDAR.

*OpenCalib* (https://github.com/PJLab-ADG/SensorsCalibration, accessed on 25 November 2022) is a calibration tool specialized in the calibration of autonomous driving systems [26]. Several sensors are supported, such as RGB cameras, LiDAR, Radio Detection And Ranging (RaDAR) and IMU. *OpenCalib* addresses the calibration of complex systems in a pairwise manner. Sensors are calibrated in pairs and the complete system is built from a compilation of several sensor pairs. As such, there are many available scripts to address each of the combinations of sensor modalities. Because different algorithms and methodologies are used for each combination of modalities, several available calibration patterns are also used according to the selected sensor modalities: chessboard, circlegrid, vertical board, AprilTag board, ArUco marker board and the round hole board [26]. The pairwise philosophy employed by *OpenCalib* results in a set of tools that calibrate specific cases. This entails special configurations, calibration patterns and labeling procedures for each case, which has the disadvantage of addressing each of the calibration problems separately, not tackling the problem using a unified and holistic approach. The UI allows for the visualization of overlapped data from different sensors. In addition, it presents movable bars which can be used to adjust calibration parameters, as seen in Figure 3.

The previously described approaches present various advantages and disadvantages. *OpenCV Camera Calibration* is by far the most simplistic of the frameworks, having a crucial stage, the data acquisition, carried out externally. This disengagement can lead to incompatibilities between the acquired data and the framework. In contrast, some approaches, such as *Kalibr* and *camera_calibration*, carry out this procedure automatically. Although this increases the ease of use of the approach, it diminishes the user’s control on this particular stage because the data cannot be collected in the most favorable instances. To tackle this, some approaches, such as *moveit_calibration* and *robot_calibration*, allow the user to choose which instance they want to collect data in. To improve this, the data labeling stage can be combined with the collection phase. This combination allows the user to verify if the data is correctly labeled before choosing to collect it. In the pose estimation stage, none of the approaches present any visual feedback. Therefore, the status of the procedure is unknown to the user, leading to a less interactive experience. It can also be detrimental when an error occurs because it’s harder to detect where the failure originates from.

*OpenCalib* is arguably the most developed approach described, and brings various features not included in other approaches. These features include the calibration of various modalities of sensors. They are carried out in a pairwise fashion, which in a complex system can be tiresome due to having to calibrate several sensor pairs. With several calibrations come several different methods, requirements and UI features. These differences can lead to a cumbersome procedure for the user.

Taking the previous points into account, it can be concluded that a calibration approach gives the user the option of collecting labeled data to provide visual feedback on the estimation stage, and to carry out a full-system calibration if needed.

Most calibration approaches are defined by creating cost functions that map the measurements of a calibration pattern from one sensor to the other, therefore optimizing the geometric transformation between the sensors. We refer to these approaches as sensor-to-sensor approaches. The problem with these classical approaches is that they are not suited to handle systems with multiple sensors. Moreover, these approaches are focused on specific sensor modalities and cannot be easily adapted to other cases. ATOM formulates the extrinsic calibration problem in such a way that the cost is computed after the pose of a single sensor and the pose of the calibration pattern, which is also estimated in the optimization. Therefore, the breakthrough of ATOM is to view the calibration in a sensor-to-pattern paradigm, which facilitates the introduction of novel modalities and also scales up well. The second critical component of ATOM is the usage of atomic transformations in the optimization procedure. Atomic transformations are non-aggregated geometric transformations. Their usage ensures that the methodology is general and applicable to sensor-to-sensor, sensor-in-motion and sensor-to-frame calibration problems.

ATOM is a methodology that was proven to accurately calibrate several robotic systems. Additional details on the methodology itself are provided in [1], and usage cases of varied robotic systems can be read in [14,15,16,17]. This paper is focused not on the calibration methodology of ATOM, but rather on the support functionalities that create an integrated solution for the calibration of robotic systems. The ATOM Calibration Framework also provides, in addition to the methodology, the visualization and interactive functionalities required to follow through the calibration of a robotic system from start to finish.

## 3. Proposed Framework

As stated in Section 1, a calibration procedure requires more than a formulation, needing to be accompanied by a framework. As such, we view ATOM not only as a novel calibration method but also as a complete calibration framework (https://github.com/lardemua/atom, accessed on 25 November 2022). To provide a set of calibration tools that are easily used by the community, ATOM is integrated into ROS, which is the standard library for the developm. Note that this is the last chance to make textual changes to the manuscript. Some style and formatting changes may have been made by the production team, please do not revert these changes.

**Figure 4:**
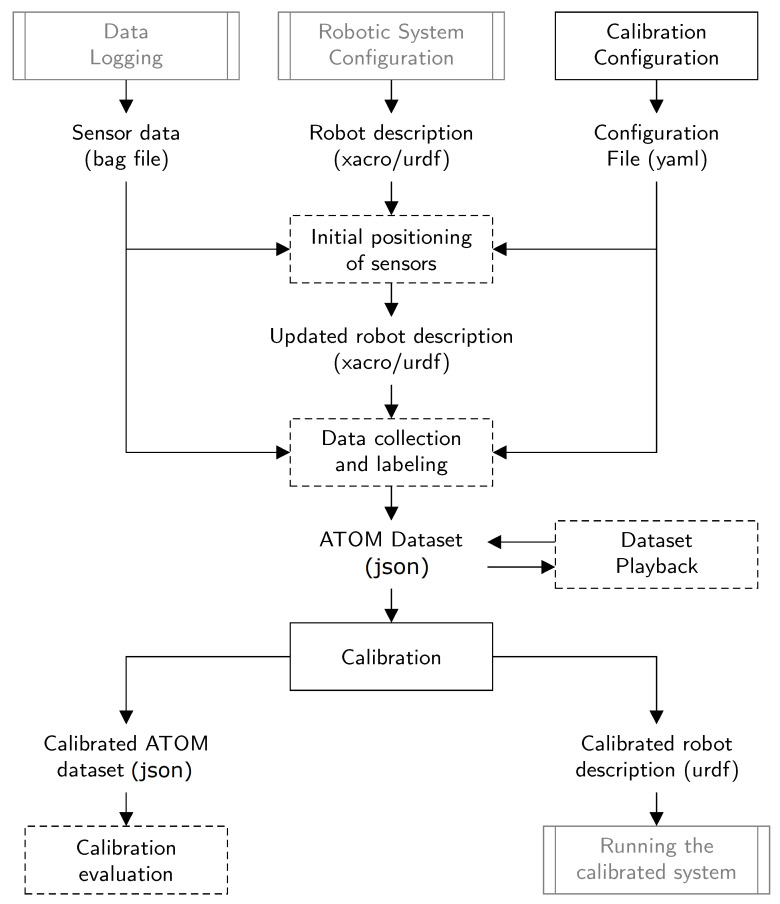
The software architecture for the ATOM calibration framework is composed of modules external to ATOM (presented in grey) and modules internal to ATOM (presented in black). Some modules are not mandatory and are presented inside a dotted bounding box.

Figure 4 provides a schematic of the proposed calibration framework. To perform a system calibration, ATOM requires data logged from the system’s sensors, provided in the format of an ROS bag file, and also a description of the configuration of the system, as given in ROS *xacro* (http://wiki.ros.org/xacro, accessed on 25 November 2022) or *urdf* (http://wiki.ros.org/urdf, accessed on 25 November 2022) formats. Note that these two requirements are external to ATOM and are not considered a heavy burden since the typical configuration of robotic systems in ROS already includes them. In ATOM, the calibration procedure is structured in five phases, which occur in sequence: (1) calibration configuration, (2) initial positioning of the sensors (not mandatory), (3) data collection and labeling, (4) dataset reviewer (not mandatory) and (5) calibration. Each of these will be described in detail in the next sections.

### 3.1. Multi-Modal Test roBot

**Figure 5:**
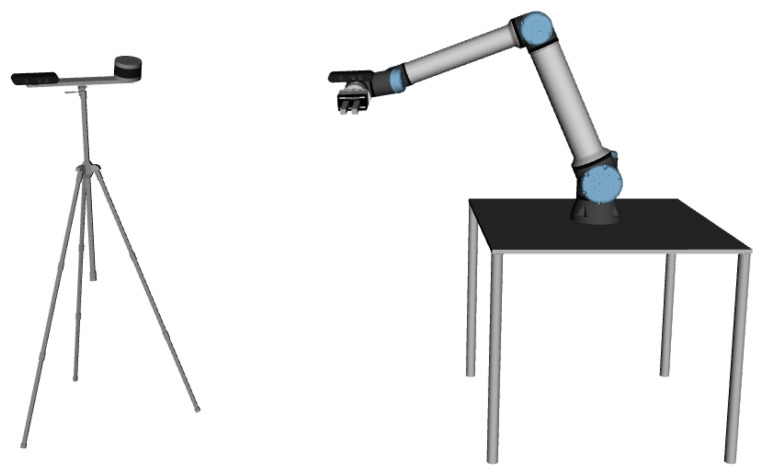
The Multi-Modal Test roBot (MMTBot), a simulated robotic system containing, from left to right in the figure: an RGB camera and a 3D LiDAR mounted on a tripod, and a second RGB camera assembled on the end effector of a robotic manipulator [1].

To better illustrate the concepts that will be detailed ahead, a robotic system called Multi-Modal Test roBot (MMTBot) will be used (https://github.com/miguelriemoliveira/mmtbot, accessed on 25 November 2022) [1]. MMTBot is a conceptual and simulated robot designed to test the performance of advanced calibration methodologies. The system contains the following sensors: *lidar*, a 3D LiDAR mounted on the left side of a tripod; *world camera*, an RGB camera mounted on the right side of that same tripod; and a *hand camera*, a second RGB camera assembled on the en effector link of a robotic manipulator, which is mounted on a table. The complete system is presented in Figure 5.

MMTBot is a robotic system which combines RGB and LiDAR modalities. Moreover, one of the RGB cameras (*hand camera*) is assembled on the end effector of the robotic manipulator, which brings a hand–eye calibration problem into the system. Because MMTBot is simultaneously a multi-sensor, multi-modal, sensor-to-sensor as well as a sensor-to-frame calibration problem, no solution in the literature can conduct the complete, simultaneous calibration of this system.

### 3.2. Calibration Configuration

The configuration defines the parameters which will be used throughout the calibration procedure, from the definition of the sensors to be calibrated to a description of the calibration pattern. These transformations are combined through the use of the topological information contained in a transformation tree, which is generated in ROS from the information from an *urdf* or *xacro* file. To calibrate the system, additional information must be provided to define which atomic transformations will be optimized during the calibration procedure. Also, a description of the calibration pattern must be provided to ensure correct detection and labeling. All this information is defined in a calibration configuration file.

### 3.3. Initial Positioning of Sensors

Since the calibration procedure is formulated as an optimization problem, the initial value of the parameters to be estimated is determinant of the outcome of the optimization. When the initial parameter values are far from the optimal parameter configuration (the solution), it is possible that the optimization converges into local minima, thus failing to find adequate values for the parameters. This problem is tackled by ensuring that the initial values contain a plausible first guess. Although recommended, this step is not mandatory if the user considers the information described inside the *urdf* or *xacro* file to be accurate.

**Figure 6:**
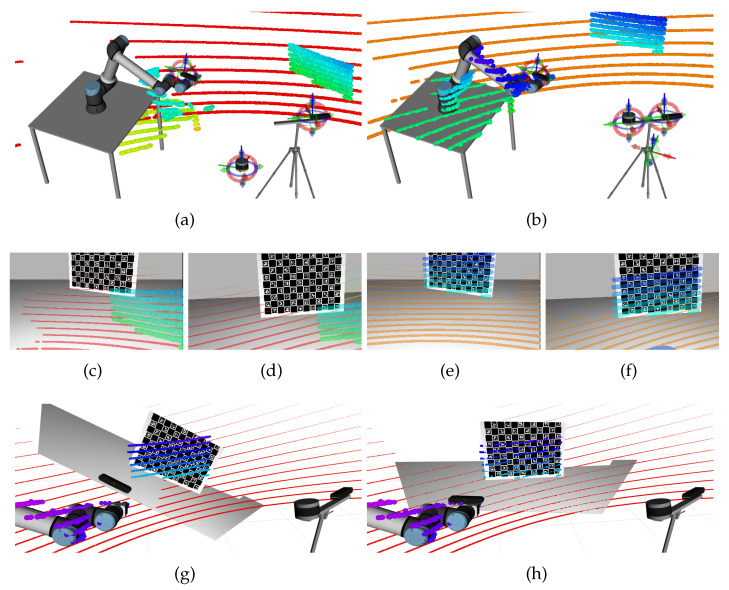
Setting an initial estimate for the sensor poses using RViz interactive markers. Top two rows: Inaccurate (left side) vs accurate (right side) positioning of the *3d LiDAR* sensor. On the left side, the 3D points from the pattern (the blue-green spheres in (**a**)) do not align well with the pattern of the images of the *world camera* (**c**) and the *hand camera* (**d**) images. The right side shows how an accurate positioning of the sensor aligns the projection of the 3D LiDAR measurements (blue spheres in (**b**)) with the pattern in the images of the *world camera* (**e**) and the *hand camera* (**f**). Bottom row: inaccurate (left) vs accurate (right) positioning of an RGB-D camera. In (**g**), the point cloud produced by the RGB-D camera (points textured with real colors) is misaligned with LiDAR point cloud (red to blue spheres). In (**h**), a correct positioning of the RGB-D camera produces a good alignment between point clouds.

The goal of a calibration operation is to find the position of the sensors, which in our approach is accomplished through the estimation of atomic transformations. Hence, the first type of parameters to initialize are these atomic transformations which account for the position of the sensors. To accomplish this, ATOM provides an interactive tool that parses the calibration file and creates a 6-DOF interactive marker associated with each sensor, which overlays on top of the ROS based robot visualization in RViz. The sensors are positioned by dragging the interactive markers, which is a simple method to easily generate plausible first guesses for the poses of the sensors. The process is very intuitive because visual feedback is provided to the user by the observation of the 3D models of the several components of the robot model and how they are spatially arranged. For example, despite not knowing the exact metric value of the distance between the *world camera* and the *3d LiDAR*, the user will know that both are more or less at the same height and not more than a meter away from each other. These non-metric, symbolic spatial relationships, in which humans are very proficient, are very useful to generate plausible first guesses. Moreover, the system provides several other visual hints that make the positioning of the sensors an intuitive process. One of these is the ability to visualize how well the data from several sensors aligns. Figure 6 shows an example. On the left side of the figure, an inaccurate positioning of the LiDAR sensor (a) leads to a misaligned projection of the blue spheres on the images (c) and (d). Conversely, an adequate positioning of that same sensor produces a good alignment between the blue spheres in (b) and the patterns in the images (see (e) and (f)). It is also possible to see that the alignment between the LiDAR measurements and the physical objects (e.g., table and manipulator) is much better in (b) when compared to (a). The bottom row of Figure 6 shows the alignment between two point clouds, the first produced by the *LiDAR* and the second by an RGB-D hand camera (which is used here just for the sake of example). Here, it is also very intuitive to realize that the alignment between point clouds is much better when the camera is adequately positioned: the alignment in (h) is better than in (g).

### 3.4. Data Collection

To ensure that the calibration is representative, and thus accurate, several views of the calibration pattern should be used.

In the case of systems containing more than one sensor, it is very common that the data coming from the sensors is streamed at different frequencies. As such, a synchronization mechanism is required to ensure that the information contained in the collection is consistent. This is not a trivial problem to address because the data is not synchronized, i.e., there is never an instant in which all sensors collect data.

This problem is solved through the use of a methodology that ensures the synchronization, assuming the scene has remained static for a *long enough period*. In static scenes, the problem of data de-synchronization is not observable, which warrants the assumption that for each captured collection the sensor data is *adequately* synchronized. Assuming it is possible to establish an upper bound to the maximum time difference between any data streaming from the robotic system, it is possible to assume that all data messages are synchronized, if the scene has remained static for a period longer than that upper bound. Thus, the methodology establishes that it is the responsibility of the user to ensure that the scene has remained static for a given minimum period before triggering the saving of a collection.

Other approaches use a similar methodology, in particular by holding the pattern with a tripod (which ensures it does not move) before collecting each image. This approach is used in multi sensor calibration frameworks [21,25], and also in RGB-D camera calibration procedures (https://github.com/code-iai/iai_kinect2, accessed on 25 November 2022).

We refer to the set of collections obtained from a given robotic system as an ATOM dataset. It contains a copy of the calibration configuration file and high-level information about each sensor, such as the sensor topological transformation chain, extracted from the transformation tree. In addition, there is also specific information for each collection, i.e., sensor data and labels, as well as values of atomic transformations. It is important to note that the set of collections should contain a sufficiently varied set of pattern poses. As such, collections should preferably have different distances and orientations with regard to the calibration pattern, so that the calibration returns more accurate results. Also, if the robotic system contains moving components, the dataset should include several poses of the system. Empirically we have found that 20 to 30 collections are a sufficient number to achieve accurate calibrations.

### 3.5. Data Labeling

The labeling of data refers to the annotation of the portions of data that view the calibration pattern. The labeling procedure is executed for the data of each sensor so that all collections have labels that correspond to the raw sensor data. Labeling can be automatic, semi-automatic or even manual in some cases. The information that is stored in a given label depends on the modality of the sensor which is being labeled.

RGB modality labels consist of the pixel coordinates of the pattern corners detected in the image, and are labeled using one of the many available image-based chessboard detectors [29]. Our system is also compatible with ChArUco boards, which have the advantage of being detected even if they are partially occluded [30]. Also in this case we make use of off-the-shelf detectors [31,32].

**Figure 7:**
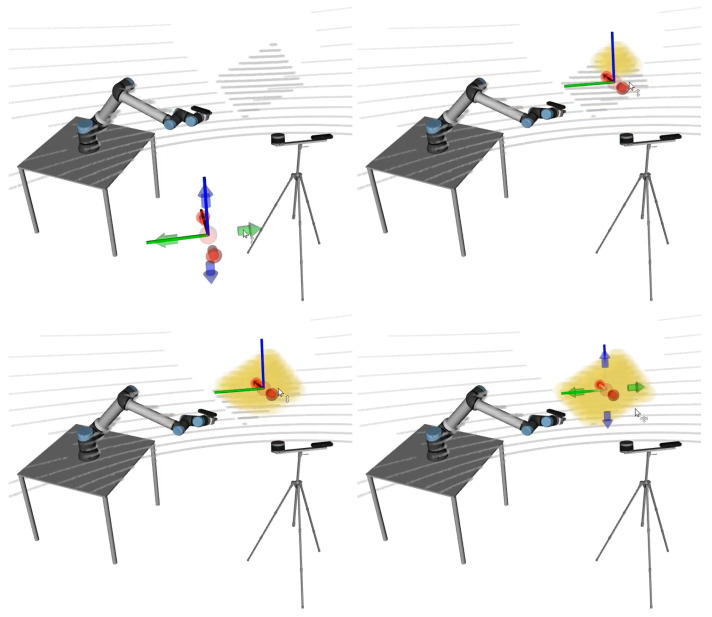
Semi-automatic LiDAR labelling procedure. In a span of about 5 s, the user drops the interactive marker close to the pattern, and the system starts tracking it. Yellow spheres indicate *lidar* measurements considered as pattern detections.

The structure of the labels is more complex in the case of the LiDAR modality. As discussed in Oliveira et al. [1], two different types cost functions are used in the optimization of LiDAR sensors: orthogonal and longitudinal. The range of measurements that belong to the pattern are referred to as the set of detections D. The *LiDAR* directly produces 3D point coordinates, so it is straightforward to obtain the 3D coordinates of a given detection *d*. The difficulty lies in finding the detections D that belong to the pattern, which is a subset of the complete set of LiDAR measurements. We propose to achieve this using a semi-automatic approach. The intervention of the user is required to set a 3D point used as the seed of a region-growing algorithm. Then, starting at the seed point, and assuming that the pattern is physically separated from the other objects in the scene, the algorithm searches in the set of *LiDAR* measurements for *close enough* points, and includes these into the set of pattern detections D, which are used as seed points in the next iteration. The process is repeated until no *close enough* points exist. The set of points traversed in the search constitutes the set of detections D. Figure 7 shows a representation of this process.

**Figure 8:**
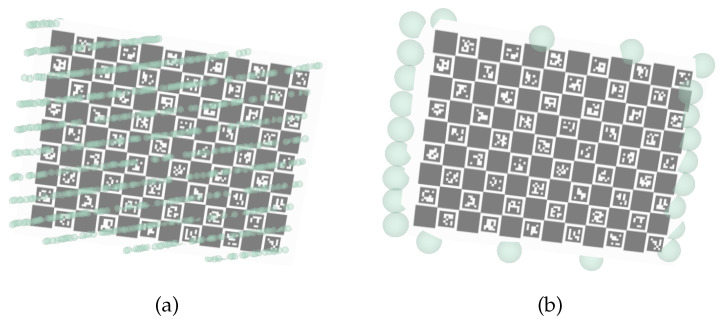
Labeling of 3D LiDAR data: (**a**) LiDAR points labeled as detections; (**b**) LiDAR points labeled as boundary points.

The second component of the LiDAR cost function is the longitudinal evaluation. For this, it is necessary to retrieve the set of measurements labeled as boundaries, which we denote as B∈D, to recover the 3D coordinates of these points. Once again, the challenge is not to find the 3D coordinates but rather which points, from the set D, are boundary points, meaning they are measuring the physical boundaries of the pattern board. The solution for this is to use a spherical parameterization to represent the point coordinates of $x_{d}\forall d\in D$. Let $s_{d}=\left[\rho_{d},\theta_{d},\varphi_{d}\right]$ represent the spherical coordinates of the detection *d*. A 3D LiDAR generally contains a much larger horizontal angular resolution, i.e., for the θ angle, when compared with the vertical angular resolution, which corresponds to the φ angle. 3D *lidars* are said to have scan *layers*, where the vertical angle is the same for a set of measurements spanning all available horizontal angles. The set of measurements corresponding to a scan layer with angle $\varphi_{l}$, denoted as $\left\{s_{\left[d,\varphi_{l}\right]}\right\}$, are extracted as follows:

(1) $$\left\{s_{\left[d,\varphi_{l}\right]}\right\}=\left\{s_{d}:\varphi_{l}-\frac{\Delta \varphi }{2}\leq \varphi_{d}<\varphi_{l}+\frac{\Delta \varphi }{2}\right\},\;\forall d\in D,$$

where Δφ is the vertical angular resolution of the *LiDAR*. For each set of detections in a layer, the left and right boundary points, denoted as $\left\{s_{\left[b,\varphi_{l}\right]}\right\}$, are extracted by searching for the measurements that have the smallest and largest value of horizontal angle θ:

(2) $$\begin{array}{c} \left\{s_{\left[b,\varphi_{l}\right]}\right\}=\left\{s_{\left[d,\varphi_{l}\right]}:\theta_{\left[d,\varphi_{l}\right]}=\max\left(\left\{\theta_{\left[d,\varphi_{l}\right]}\right\}\right)\;\;\vee \theta_{\left[d,\varphi_{l}\right]}=\min\left(\left\{\theta_{\left[d,\varphi_{l}\right]}\right\}\right)\right\},\;\forall d\in D, \end{array}$$

and finally, the complete list of boundary points B of the calibration pattern, is given by putting together the boundary points detected for each scan layer. Figure 8 shows an example of the LiDAR labeling procedure. The labeled boundary points are signaled by the large green spheres in (b).

The labeling procedure for the depth modality is semi-automatic. The user must click in the middle of the pattern in the depth image displayed in RViz. This pixel coordinate provided by the user is the seed point used in a flood-fill algorithm carried out in the depth image. Assuming that the pattern is physically distant from objects, the flood fill will propagate until the boundaries of the pattern, at which point the jump to pixels containing depth information from other objects will be too large and above the predefined threshold. After the reconstruction of the pattern area in the image, there is a sub-sampling procedure to obtain the pattern points, and a convex hull operation that extracts the boundary points.

**Figure 9:**
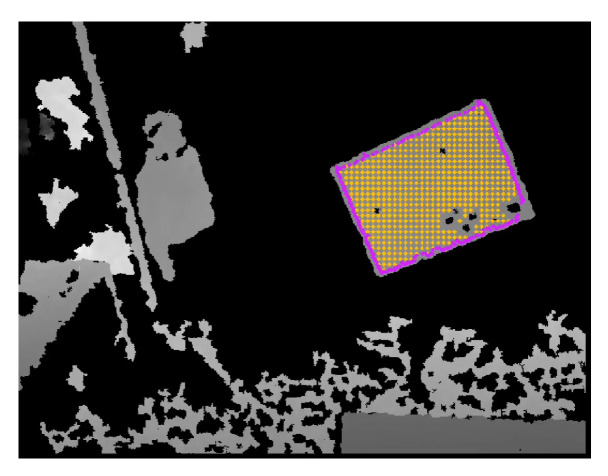
Labeling of depth camera data, with yellow pixels as detections and magenta pixels as boundary pixels.

The depth sensor modality yields a representation for the labels similar to the one previously described for the LiDAR modality [17]. It contains pattern points (magenta points in Figure 9), and also boundary points (yellow points in Figure 9). The boundary points differ from the depth to the LiDAR modalities because LiDAR sensors have a small vertical angular resolution with regard to the horizontal resolution. This leads to the annotation of only two horizontal boundary points per vertical LiDAR layer, as defined in Equations (1) and (2), and displayed in Figure 8.

### 3.6. Dataset Reviewer

The automatic and semi-automatic labeling procedures described in the previous sections are designed to facilitate the calibration procedure. Nonetheless, when the goal is to obtain very accurate calibrations, it is recommended that an experienced user reviews the automatically produced labels. The dataset reviewer module was created for this purpose. It parses an ATOM dataset and displays in RViz a collection with the 3D model of the robotic system, the corresponding sensor data and the produced labels overlayed on top of it. The user can navigate all collections in the dataset, reviewing each separately. This allows for an inspection of each collection, which makes it easy to identify errors produced by the automatic labeling procedures. It is also possible to delete mislabels and to interactively add new labels. This enables the user to use some of the automatically produced labels, or to discard them and generate all labels manually.

From the sensor modalities supported in ATOM, only the RGB camera labels are not supported by the ATOM dataset reviewer. The reason is that the detection of the pattern in RGB images is very robust and never requires user intervention.

**Figure 10:**
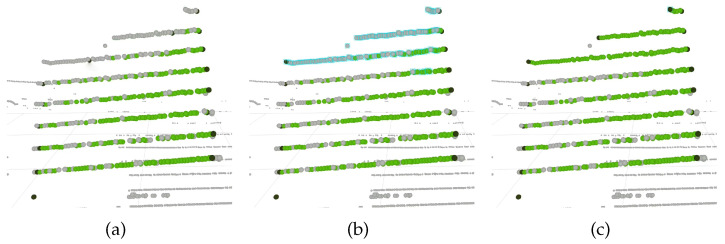
Dataset review and correction of lidar sensors: (**a**) inaccurate labeling; (**b**) selection of inaccurately labeled points; (**c**) accurate labeling after manual correction.

For the LiDAR modality, the system shows the two distinct label classes, pattern points and boundary points (see Section 3.5) with green color and dark color, respectively, as shown in Figure 10. Each 3D point corresponds to a LiDAR measurement and can be selected by the user. Then, it is possible to define the action to be carried out on the selected points: delete, annotate as pattern points or annotate as boundary points. Figure 10a–c shows a sequence of images taken during a labeling correction procedure: in (a), the labels produced by the automatic procedures are not accurate, as the upper layers of the LiDAR, which are clearly observing the pattern, are not included as pattern points. In addition to this, the second and third LiDAR layers (from top to bottom) contain incorrectly labeled boundary points; in (b) the user selects the upper layers. The selection is signaled by blue bounding boxes around each point; then, in (c), the user first deletes the labels of the selection (which solves the incorrectly labeled boundary points), and then labels the selected points as pattern points. This selection is followed by three different relabeling options: label as a detection, label as a boundary point or label as unlabeled.

**Figure 11:**
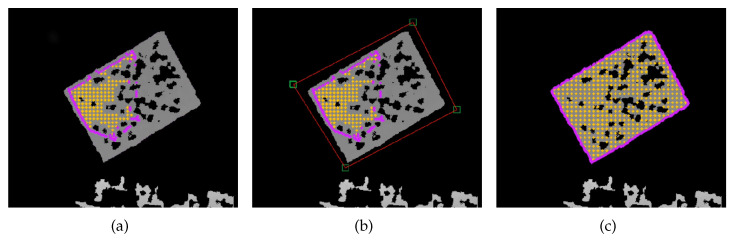
Dataset review and correction of depth sensors: (**a**) inaccurate labeling produced by automatic procedure; (**b**) definition of a polygon around the calibration pattern; (**c**) extraction of accurate labeling from the polygon.

On the depth modality, the pattern points and boundary points (see Section 3.5) are presented in yellow and magenta, respectively, in Figure 11. The depth image is presented in RViz and the user can click on it to select a point. This point is represented visually with a green square surrounding it. Then, if a second point is selected, a red line connects both. This process continues to connect points whenever a new one is placed. If the user clicks near the first point, a polygon is formed from the connecting lines. All depth measurements inside the polygon are considered to be inside the pattern. Then, we process this data to sub-sample and remove pixel coordinates that correspond to invalid range measurements. A convex hull operation is used to generate the boundary points. Figure 11a–c shows a sequence of images representing the depth labeling correction procedure: in (a), it is clear that the labels generated from the semi-automatic labeling procedure are inaccurate; in (b), the user creates a polygon surrounding the pattern by clicking on four different points in the image, which define the corners of the red polygon; in (c), the new labels for the depth image are extracted from the polygon, as described above.

### 3.7. Calibration

The vast majority of calibration approaches do little more than printing some information on the screen to display the results of the calibration. ATOM provides a great deal of visual feedback, not only at the end of the calibration but also throughout the procedure.

To accomplish this goal, the information stored in an ATOM dataset is published into the ROS ecosystem. The system’s configurations for all collections are simultaneously conveyed to ROS, as if they had occurred all at the same time which, not being the case, is the framework under which the calibration procedure operates. Collisions in topic names and reference frames are avoided by adding a collection-related prefix to each designation. Also, the original transformation tree is replicated for each collection and those subtrees are connected so that it is possible to display them together.

**Figure 12:**
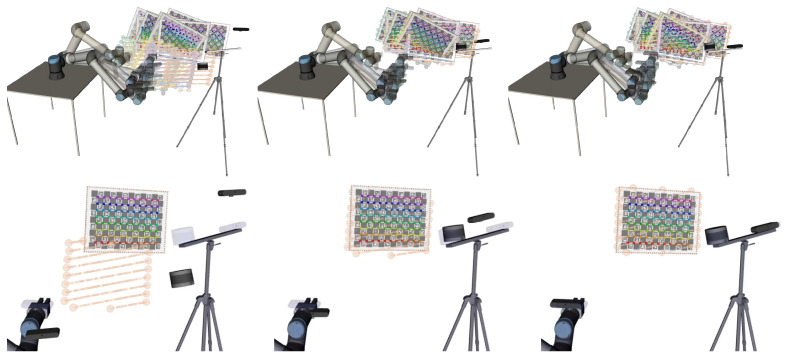
Visualizing the evolution of a calibration procedure from the initial estimate (**left**) to the solution (**right**). The top row shows all collections used in this calibration (different colors for each collection). The bottom row shows a single collection and, in transparent mode, ground truth poses of the sensors (known because this dataset was generated in simulation).

Figure 12 shows an example of the visualization of a calibration procedure. It is possible to simultaneously visualize the collections that are being used in the calibration (top row) or to select which collections are displayed (bottom row). The ground truth poses of the sensor appear in transparent mode. The ground truth poses are known only because MMTBot is a simulated robotic system. For real robotic systems, there is no ground truth information (the transparent mode is used to show the initial pose of the sensors). It is possible to observe that, as the optimization progresses (from left to right in Figure 12), the sensors move towards the ground truth pose (transparent mode), which means that the optimization is converging towards the optimal solution.

The integration with ROS provides straightforward access to many other interesting functionalities. For example, it is possible to visualize images with the reprojected pattern corners, to display the robot meshes, the position of the reference frames and so forth.

## 4. Results

As stated in Section 1, this article describes the calibration framework behind ATOM. In contrast with the calibration methodology, it is not easy to produce quantitative evaluations for the ATOM calibration framework. However, we provide extensive qualitative demonstrations of the stages of the framework, as well as the results of a survey meant to illustrate users’ opinions on calibration approaches as well as how users compare ATOM with other frameworks.

### 4.1. Qualitative Demonstrations of ATOM

This subsection includes demonstrations of the calibration of several robotics systems of diverse configurations, aims to describe the user interface of the ATOM calibration framework, and shows the advantages this framework brings to the user experience.

**Figure 13:**
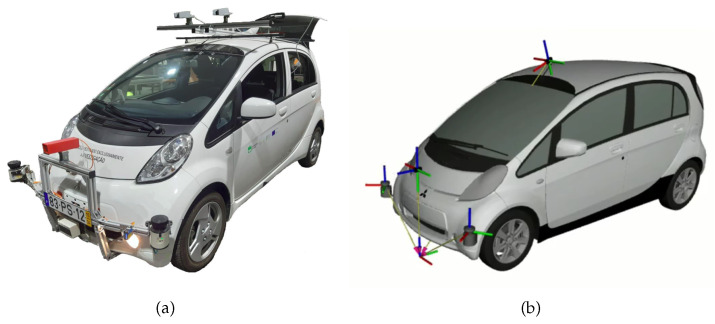
AtlasCar2 is an autonomous vehicle with two RGB cameras and two 2D LiDAR, besides other sensors not tackled here. (**a**) represents the real system and (**b**) represents the simulated system.

**Figure 14:**
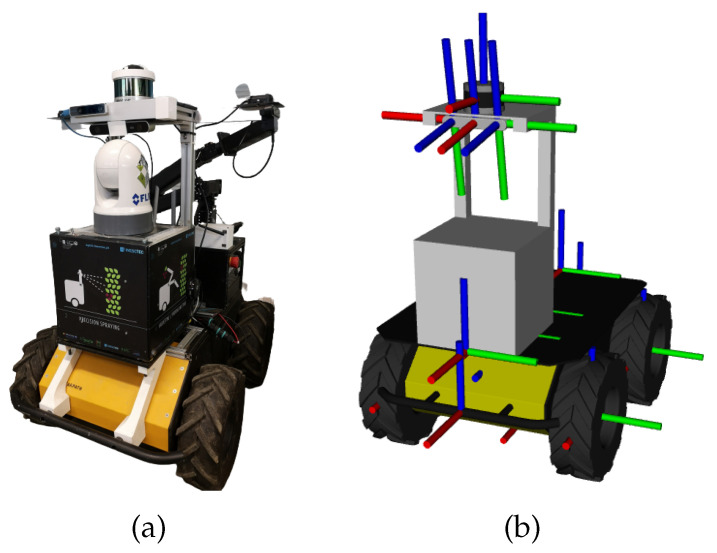
AgRob V16 is an Automated Guided Vehicle (AGV) used for agronomic purposes. It consists of a 3D LiDAR and a stereo camera system. (**a**) represents the real system and (**b**) represents the simulated system.

**Figure 15:**
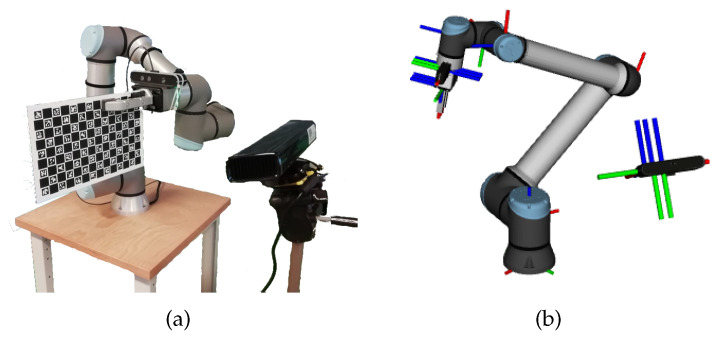
Iris UR10e is a robotic system comprised of a robotic manipulator with two RGB cameras, one fixed to the end-effector of the robotic manipulator and another mounted on a tripod. (**a**) represents the real system and (**b**) represents the simulated system.

**Figure 16:**
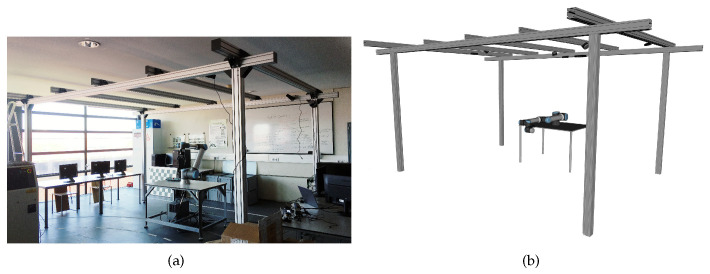
LARCC is a collaborative cell with a robotic manipulator, three RGB cameras, three 3D LiDARs and one depth camera [17]. (**a**) represents the real system and (**b**) represents the simulated system.

The results are presented in five distinct robotic systems. The MMTBot is a robotic system that contains two RGB cameras and one 3D LiDAR, as already described in section Section 3.1. This system configuration can be seen as a sensor-to-sensor, as well as a sensor-to-frame calibration problem. The AtlasCar2 [16,33,34] is a prototype of an autonomous vehicle equipped with several sensors, namely two RGB cameras and two 2D LiDARs. This system configuration can be seen as a sensor to sensor calibration problem. Figure 13 presents the AtlasCar2, both the real system (a) as well as the digital model used in the framework (b). The AgRob V16 [15] (Figure 14) is an agronomic vehicle with a 3D LiDAR and a stereo camera system. In this case, we are dealing with a sensor-to-sensor calibration problem. The Iris UR10e [14] (Figure 15) is a system composed of a robotic arm containing two RGB cameras, one fixed to the end effector of the robotic manipulator and the other mounted on a tripod. This system is a hand–eye calibration system, which is simultaneously a sensor in motion and sensor to sensor calibration problem. LARCC [17] (Figure 16) is a collaborative cell equipped with a robotic manipulator, three RGB cameras, three 3D LiDARs and one depth camera. This system is a sensor-to-sensor calibration problem, with a large number of sensors and modalities, which makes the problem of calibrating such a system very challenging.

Table 1 shows the calibration procedures described in Figure 4 for each of the robotic systems. Results show that the framework operates well on all of the tested systems. It supports the calibration configuration, initial positioning of sensors, data collection and labeling, dataset reviewer and the calibration itself. All these systems together represent a large spectrum of calibration configurations, from-sensor-to-sensor, to sensor-in-motion and to sensor-to-frame. In addition, there is also a large variety in the number of sensors and their modalities. This is a statement of the general nature of both the ATOM methodology as well as the flexibility of the supporting framework.

### 4.2. User Experience Survey

**Figure 17:**
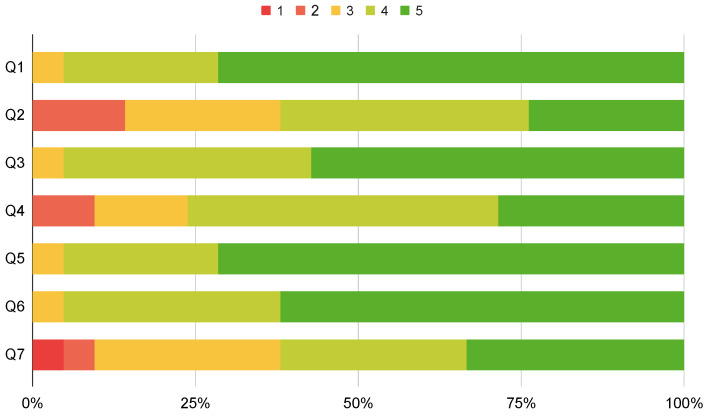
Responses to questions **Q1–Q7**. Likert scale from 1 (strongly disagree) to 5 (strongly agree). **Q1**—The calibration is a crucial component of a robotic system. **Q2**—A calibration approach should be interactive, since that helps to produce better calibrations and understand why something went wrong. **Q3**—It is helpful to have the visualization of different calibration components. **Q4**—The user should have detailed control of the calibration procedure, for example, being able to configure the calibration or to inspect and correct labels. **Q5**—The ability to calibrate systems with more than two sensors is important in a calibration approach. **Q6**—The ability to calibrate systems with several sensor modalities is important in a calibration approach. **Q7**—It is not ideal to calibrate complex, multi-sensor, multi-modal robotic systems with different approaches for each combination of sensors and modalities.

**Figure 18:**
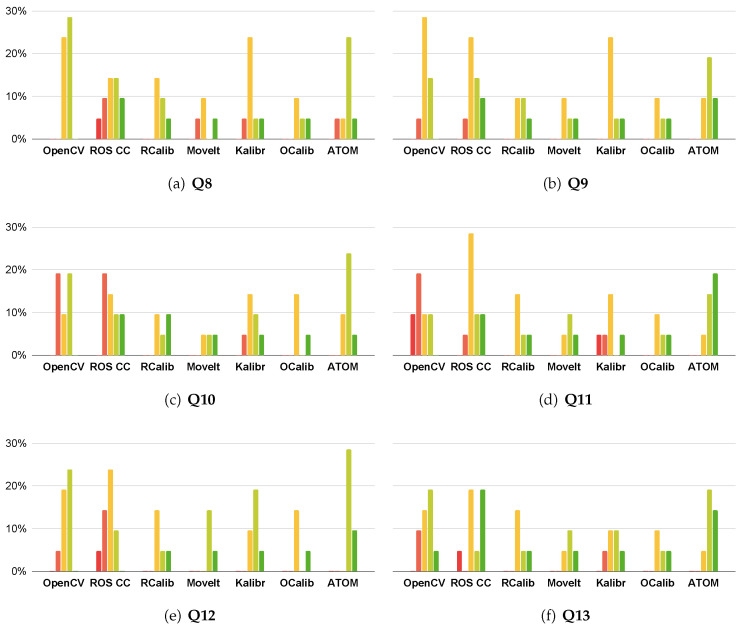
Responses to questions **Q8–Q13**. Likert scale from 1 (strongly disagree) to 5 (strongly agree). Red represents 1, orange represents 2, yellow represents 3, light green represents 4 and green represents 5. **Q8**—It is easy to calibrate a system using this approach. **Q9**—This approach is intuitive to use. **Q10**—The calibration approach provides useful information to understand each of the calibration procedures. **Q11**—The interface is helpful. **Q12**—The calibration approach produces accurate extrinsic parameters. **Q13**—After calibration, it is straightforward to integrate the results (the output) of the calibration into the robotic system.

**Figure 19:**
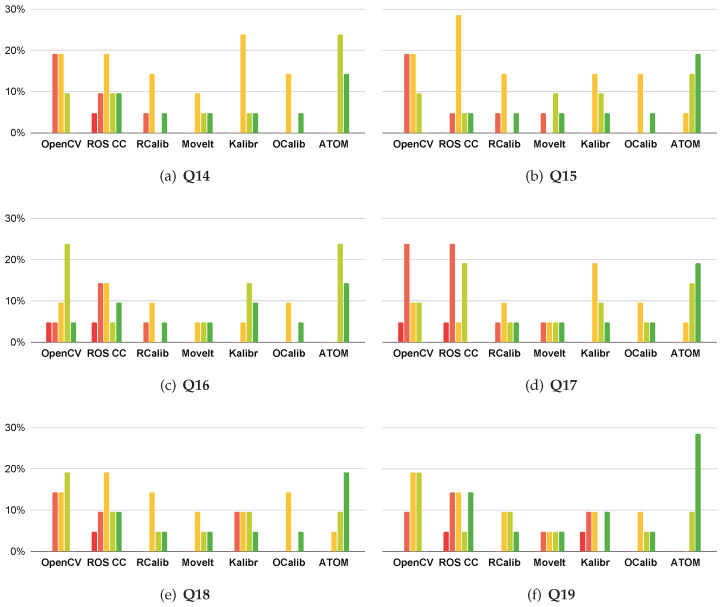
Responses to questions **Q14–Q19**. Likert scale from 1 (strongly disagree) to 5 (strongly agree). Red represents 1, orange represents 2, yellow represents 3, light green represents 4 and green represents 5. **Q14**—The collection of data for calibration is well solved by this approach. **Q15**—The labeling of data for calibration is well solved by this approach. **Q16**—The estimation of extrinsic parameters is well solved by this approach. **Q17**—The evaluation of the result of the calibration is well solved by this approach. **Q18**—The approach successfully calibrated my robotic system. **Q19**—I am likely to use this approach again.

To better understand the public opinion behind calibration approaches, a survey was conducted. This survey consists of nineteen questions, and splits into three different groups: The first group (**Q1–Q7**) assesses which qualities are important for a good calibration procedure (see Figure 17); The second group (**Q8–Q13**) compares different calibration approaches (see Figure 18); The third (**Q14–Q19**) focuses on the components of a calibration procedure (see Figure 19). These questions follow a Likert level format, with answers ranging from 1 (strongly disagree) to 5 (strongly agree) [35]. The target audience for this survey are robotic specialists who have already calibrated a robotic system. To reach this audience, the survey was posted in an ROS specialized forum (https://discourse.ros.org/, accessed on 25 November 2022), and sent to a more general robotics mailing list (http://robotics-worldwide.org/, accessed on 25 November 2022). We obtained a total of twenty-one answers.

The first group presents questions on general characteristics that should be present in a calibration approach. Each question contains a statement to be rated by the user. Figure 17 shows the results for questions **Q1** through **Q7**.

Results for **Q1** show that the large majority of users view the calibration as a crucial component of a robotic system. **Q2** is not as consensual. In our opinion, this is because many users would prefer a fully automated calibration procedure. However, we believe that the most important factor is accuracy, which generally requires some form of user intervention. **Q3** and **Q4** show that the users value the visualization and detailed control of a calibration procedure. **Q5** and **Q6** prove that robotic systems require more complex calibration solutions since users view the need for multi-sensor and multi-modal functionalities as relevant. However, there is no consensus on how to approach the calibration of complex robotic systems. As shown in **Q7**, some users would prefer a single approach, while others see a set of specialized calibration approaches as better.

The second group of questions (**Q8–Q13**) asks the users to assess several calibration approaches comparatively. As discussed in Section 2, these approaches are: OpenCV Camera Calibration (**OpenCV**) [18], ROS Camera Calibration (**ROS CC**), Robot Calibration (**RCalib**), MoveIt Calibration (**MoveIt**) [19,20], Kalibr (**Kalibr**) [21,22,23,24,25], OpenCalib (**OCalib**) [26] and ATOM (**ATOM**). Results are presented in Figure 18. In **Q8**, OpenCV, ROS Camera Calibration and ATOM are the approaches that are reportedly easier to use. Note that while ATOM can calibrate complex robotic systems with a large number of sensors and several sensor modalities, the other two approaches (OpenCV and ROS Camera Calibration) are limited to pairwise RGB systems. **Q9** focuses on how intuitive the approach is. ATOM was reported as the most intuitive calibration framework. We believe this is due to ATOM’s procedures being often accompanied by visualization and interactive functionalities. **Q10** measures the information provided by the calibration framework. OpenCV and ROS Camera Calibration exhibit the poorest performance, possibly due to the fact they do not convey information to the user in many calibration stages, such as the estimation of extrinsic parameters. ATOM and Robot Calibration provide useful information. In **Q11**, OpenCV has the least helpful interface, which is understandable since it is an API and not an application. ATOM and MoveIt Calibration are the approaches with the most helpful interface, both based on RViz. In **Q12**, users consider ROS Camera Calibration as the less accurate calibration procedure. ATOM is reported to be accurate by all users who have experienced it. In **Q13**, ROS Camera Calibration and ATOM present the most easy to integrate outputs. Both use ROS readable formats.

The third group of questions (**Q14–Q19**) focuses on how each calibration approach tackles the typical stages of a calibration: data collection, data labeling and estimation of calibration parameters. Results are presented in Figure 19. In **Q14**, OpenCV has no data collection tool, so it does not solve this calibration stage. ATOM is reportedly the approach that best solves the data collection stage. In **Q15**, OpenCV presents the worst labeling feature, possibly due to the lack of a procedure to verify the automatically generated labels. ATOM is the best calibration approach regarding the labeling of data, validating the proposed methodology of combining the collection and labeling stages. In **Q16**, most approaches solve the calibration of extrinsic parameters. Nonetheless, ATOM presents the best results in this regard. We believe this is due to an extensive visualization of the calibration procedure, something no other approach does. In **Q17**, users highlighted OpenCV and ROS Camera Calibration because these frameworks do not include evaluation procedures. ATOM, on the other hand, provides extensive evaluation procedures which the users valued. In **Q18**, ATOM has the highest number of successful calibrations. Also, no user disagreed with the statement, meaning that ATOM satisfactorily solved all calibration problems it was presented with. In **Q19**, ATOM is the clear front runner since all users who have tried it intend to use it again.

## 5. Conclusions

In this paper, a novel calibration framework designed specifically to support the ATOM methodology is proposed. The framework explicitly addresses all three components of a calibration procedure: data acquisition, data labeling and sensor pose estimation. We propose a system architecture that tackles those components and provides a detailed user interface to support each, as shown in Figure 4.

The first stages consist of describing the robotic system to be calibrated and configuring the calibration. Using an interactive tool, it is possible to provide plausible first guesses of the sensor poses. Then, the stage for collecting data is carried out. We propose that the collection of data is coupled with the labeling of data because the user is best informed of when a collection should be extracted if they are aware of the results of labeling in real-time. The ATOM calibration framework also supports the navigation through the collection of an ATOM dataset and the correction of the labels. Finally, the optimization of extrinsic parameters is supported by a detailed visualization of the system, the sensor data and the labels. At the end of this stage, an ATOM dataset is generated. This dataset can present inaccurate labels in specific collections, so the user has the ability to review every collection of the dataset.

The proposed framework has advantages when compared to other approaches. A clear one is how easy it is to set up the calibration, making the procedure straightforward to configure to any robotic or intelligent system, regardless of the number of sensors and their modalities. The initial position of the sensors is a very advantageous module because it decreases the possibility of the optimization algorithm getting stuck in local minima. Although already seen in some approaches, the junction of the data collection and the data labeling is a clear asset for our framework due to the ability to verify if the data is correctly labeled before choosing to collect it. Another unique resource in our framework is the dataset reviewer functionality, allowing the user to review and rectify the previously created labels.

Experiments cover five distinct robotic systems with different numbers of sensors and several combinations of sensor modalities. Results demonstrate the versatility and robustness of the framework, which can tackle the three distinct variants of the calibration problem: sensor-to-sensor, sensor-in-motion and sensor-to-frame.

In addition, a survey of calibration frameworks was conducted to assess which qualities are valued in a calibration framework. The survey covered various topics, such as the qualities of the calibration and the performance of the approach in the typical stages of calibration. Results show that the ATOM framework is viewed very favorably by the majority of users in comparison to other calibration approaches.

Future work includes the addition of more sensor modalities, which brings the need to add a new labeling procedure and a new reviewer functionality to the framework.

## Figures and Tables

**Table 1:** ATOM calibrated systems with demonstrative videos. All videos accessed in 12 January 2023.

System	Description	Sensors	Video	
			Calibration Stage	Link
MMTBot	Simulation with robotic arm Sensor to sensor Sensor to frame	RGB cameras (×2) 3D LiDAR	Calibration Configuration	https://youtu.be/2RTCUt2cdJY
			Initial Positioning of Sensors	https://youtu.be/oJLKTqUtZvQ
			Data Collection and Labeling	https://youtu.be/eII_ptyMq5E
			Calibration	https://youtu.be/4B3X_NsX89M
AtlasCar2	Autonomous vehicle Sensor to sensor	RGB cameras (×3) 2D LiDARs (×2)	Initial Positioning of Sensors	https://youtu.be/CnxzcQ3LZFA
			Data Collection and Labeling	https://youtu.be/BYs1-H9vh0s
			Calibration	https://youtu.be/Erl272fj6Rg
AgRob V16	AGV for agriculture Sensor to Sensor	Stereo camera 3D LiDAR	Calibration	https://youtu.be/QNEyUrZm8eg
				https://youtu.be/k5B0wRdWcpo
Iris UR10e	Hand-eye system Sensor in motion Sensor to frame	RGB cameras (×2)	Initial Positioning of Sensors	https://youtu.be/WGXb_oziUpA
			Data Collection and Labeling	https://youtu.be/lHEpRyfYVp8
			Calibration	https://youtu.be/8tz4qkvfj88
LARCC	Collaborative Cell Sensor to sensor	RGB cameras (×3) 3D LiDAR (×3) Depth camera	Initial Positioning of Sensors	https://youtu.be/i5GuqNeiXO0
			Data Collection and Labeling	https://youtu.be/x49d1jmpirA
			Depth Modality Labeling	https://youtu.be/OuLHvp95UBI
			Dataset Reviewer - Navigating	https://youtu.be/m9HZ-JcQOZg
			Dataset Reviewer - Depth	https://youtu.be/GuWEd2cENXM
			Dataset Reviewer - LiDAR	https://youtu.be/YtK-CqZ9gk8
			Calibration	https://youtu.be/TKyY5zdPTKs

## Data Availability

Not applicable.

## Abbreviations

The following abbreviations are used in this manuscript:

AGV	Automated Guided Vehicle
ATOM	Atomic Transformations Optimization Method’s
IMU	Inertial Measurement Unit
LiDAR	Light Detection And Ranging
MMTBot	Multi-Modal Test roBot
OpenCV	Open Source Computer Vision Library
RaDAR	Radio Detection And Ranging
ROS	Robot Operating System
RViz	ROS 3D Robot Visualizer
UI	User Interface
URDF	Unified Robot Description Format
UX	User Experience
SFM	Structure From Motion
SLAM	Simultaneous Localization and Mapping

## References

[1] Oliveira M., Pedrosa E., Aguiar A., Rato D., Santos F., Dias P., Santos V. (2022). ATOM: A general calibration framework for multi-modal, multi-sensor systems. Expert Syst. Appl..

[2] Weng J., Coher P., Herniou M. (1992). Camera Calibration with Distortion Models and Accuracy Evaluation. IEEE Trans. Pattern Anal. Mach. Intell..

[3] Carrera G., Angeli A., Davison A.J. SLAM-based automatic extrinsic calibration of a multi-camera rig. Proceedings of the 2011 IEEE International Conference on Robotics and Automation.

[4] Fernández-Moral E., González-Jiménez J., Arévalo V. (2015). Extrinsic calibration of 2D laser rangefinders from perpendicular plane observations. Int. J. Robot. Res..

[5] Almeida M., Dias P., Oliveira M., Santos V. (2012). 3D-2D Laser Range Finder Calibration Using a Conic Based Geometry Shape. International Conference Image Analysis and Recognition.

[6] Trejos K., Rincón L., Bolaños M., Fallas J., Marín L. (2022). 2D SLAM Algorithms Characterization, Calibration, and Comparison Considering Pose Error, Map Accuracy as Well as CPU and Memory Usage. Sensors.

[7] Martinec D., Pajdla T. Robust rotation and translation estimation in multiview reconstruction. Proceedings of the 2007 IEEE Conference on Computer Vision and Pattern Recognition.

[8] Zhuang H., Roth Z., Sudhakar R. (1994). Simultaneous robot/world and tool/flange calibration by solving homogeneous transformation equations of the form AX=YB. IEEE Trans. Robot. Autom..

[9] Tan J., An X., Xu X., He H. Automatic extrinsic calibration for an onboard camera. Proceedings of the 2013 Chinese Automation Congress.

[10] Pereira M., Silva D., Santos V., Dias P. (2016). Self calibration of multiple LIDARs and cameras on autonomous vehicles. Robot. Auton. Syst..

[11] Jiuqing W., Xu C., Shaocong B., Li L. (2018). Distributed data association in smart camera network via dual decomposition. Inf. Fusion.

[12] Daniel Herrera C., Kannala J., Heikkilä J. (2012). Joint depth and color camera calibration with distortion correction. IEEE Trans. Pattern Anal. Mach. Intell..

[13] Zhang Q., Pless R. Extrinsic calibration of a camera and laser range finder (improves camera calibration). Proceedings of the 2004 IEEE/RSJ Int. Conf. on Intelligent Robots and Systems (IROS) (IEEE Cat. No.04CH37566).

[14] Pedrosa E., Oliveira M., Lau N., Santos V. (2021). A General Approach to Hand–Eye Calibration Through the Optimization of Atomic Transformations. IEEE Trans. Robot..

[15] Aguiar A., Oliveira M., Pedrosa E., Santos F. (2021). A Camera to LiDAR calibration approach through the Optimization of Atomic Transformations. Expert Syst. Appl..

[16] Oliveira M., Castro A., Madeira T., Pedrosa E., Dias P., Santos V. (2020). A ROS framework for the extrinsic calibration of intelligent vehicles: A multi-sensor, multi-modal approach. Robot. Auton. Syst..

[17] Rato D., Oliveira M., Santos V., Gomes M., Sappa A. (2022). A sensor-to-pattern calibration framework for multi-modal industrial collaborative cells. J. Manuf. Syst..

[18] Bradski G., Kaehler A., Pisarevsky V. (2005). Learning-based computer vision with intel’s open source computer vision library. Intel Technol. J..

[19] Chitta S., Sucan I., Cousins S. (2012). MoveIt!. IEEE Robot. Autom. Mag..

[20] Rosen E., Whitney D., Phillips E., Chien G., Tompkin J., Konidaris G., Tellex S. (2019). Communicating and controlling robot arm motion intent through mixed-reality head-mounted displays. Int. J. Robot. Res..

[21] Rehder J., Siegwart R., Furgale P. (2016). A General Approach to Spatiotemporal Calibration in Multisensor Systems. IEEE Trans. Robot..

[22] Maye J., Furgale P., Siegwart R. Self-supervised calibration for robotic systems. Proceedings of the 2013 IEEE Intelligent Vehicles Symposium (IV).

[23] Grundmann M., Kwatra V., Castro D., Essa I. Calibration-free rolling shutter removal. Proceedings of the 2012 IEEE International Conference on Computational Photography (ICCP).

[24] Furgale P., Barfoot T.D., Sibley G. Continuous-time batch estimation using temporal basis functions. Proceedings of the 2012 IEEE International Conference on Robotics and Automation.

[25] Furgale P., Rehder J., Siegwart R. Unified temporal and spatial calibration for multi-sensor systems. Proceedings of the 2013 IEEE/RSJ International Conference on Intelligent Robots and Systems.

[26] Yan G., Liu Z., Wang C., Shi C., Wei P., Cai X., Ma T., Liu Z., Zhong Z., Liu Y. (2022). OpenCalib: A Multi-sensor Calibration Toolbox for Autonomous Driving. arXiv.

[27] Leung K. (2022). Hand-Eye Calibration Tutorial. https://ros-planning.github.io/moveit_tutorials/doc/hand_eye_calibration/hand_eye_calibration_tutorial.html.

[28] Schneider T. (2022). Kalibr Wiki. https://github.com/ethz-asl/kalibr/wiki/.

[29] Zhang Y., Li G., Xie X., Wang Z. A new algorithm for accurate and automatic chessboard corner detection. Proceedings of the 2017 IEEE International Symposium on Circuits and Systems (ISCAS).

[30] Jurado S., Salinas R., Cuevas F., Carnicer R. (2016). Generation of fiducial marker dictionaries using Mixed Integer Linear Programming. Pattern Recognit..

[31] Ramirez F., Salinas F., Carnicer R. (2018). Speeded up detection of squared fiducial markers. Image Vis. Comput..

[32] Hu D., DeTone D., Malisiewicz T. Deep ChArUco: Dark ChArUco Marker Pose Estimation. Proceedings of the 2019 IEEE/CVF Conf. on Computer Vision and Pattern Recognition (CVPR).

[33] Santos V., Almeida J., Ávila E., Gameiro D., Oliveira M., Pascoal R., Sabino R., Stein P. ATLASCAR-technologies for a computer assisted driving system, on board a common automobile. Proceedings of the 13th International IEEE Conference on Intelligent Transportation Systems.

[34] Santos V., Usikhin O., Madani K. (2020). ATLASCAR: A Sample of the Quests and Concerns for Autonomous Cars. International Conference on Informatics in Control, Automation and Robotics.

[35] Norman G. (2010). Likert scales, levels of measurement and the “laws” of statistics. Adv. Health Sci. Educ..

